# MITF functions as a tumor suppressor in non-small cell lung cancer beyond the canonically oncogenic role

**DOI:** 10.18632/aging.202171

**Published:** 2020-12-03

**Authors:** Yi-Jing Hsiao, Wen-Hsin Chang, Hsuan-Yu Chen, Yin-Chen Hsu, Su-Chin Chiu, Ching-Cheng Chiang, Gee-Chen Chang, Yi-Ju Chen, Chia-Yu Wang, Yan-Ming Chen, Chien-Yu Lin, Yu-Ju Chen, Pan-Chyr Yang, Jeremy J.W. Chen, Sung-Liang Yu

**Affiliations:** 1 Department of Clinical Laboratory Sciences and Medical Biotechnology, College of Medicine, National Taiwan University, Taipei, Taiwan; 2 Institute of Molecular Medicine, College of Medicine, National Taiwan University, Taipei, Taiwan; 3 Institute of Statistical Science, Academia Sinica, Taipei, Taiwan; 4 Inservice Master Program in Life Sciences, College of Life Sciences, National Chung-Hsing University, Taichung, Taiwan; 5 Division of Chest Medicine, Department of Internal Medicine, Taichung Veterans General Hospital, Taichung, Taiwan; 6 Institute of Chemistry, Academia Sinica, Taipei, Taiwan; 7 Institute of Biomedical Sciences, Academia Sinica, Taipei, Taiwan; 8 Department of Internal Medicine, National Taiwan University Hospital, Taipei, Taiwan; 9 Institute of Biomedical Sciences, National Chung-Hsing University, Taichung, Taiwan; 10 Department of Laboratory Medicine, National Taiwan University Hospital, Taipei, Taiwan; 11 Centers for Genomic and Precision Medicine, National Taiwan University, Taipei, Taiwan

**Keywords:** FZD7, metastasis, transcriptome profiling, WNT pathway

## Abstract

Microphthalamia-associated transcription factor (MITF) is a critical mediator in melanocyte differentiation and exerts oncogenic functions in melanoma progression. However, the role of MITF in non-small cell lung cancer (NSCLC) is still unknown. We found that *MITF* is dominantly expressed in the low-invasive CL1-0 lung adenocarcinoma cells and paired adjacent normal lung tissues. *MITF* expression is significantly associated with better overall survival and disease-free survival in NSCLC and serves as an independent prognostic marker. Silencing *MITF* promotes tumor cell migration, invasion and colony formation in lung adenocarcinoma cells. In xenograft mouse model, *MITF* knockdown enhances metastasis and tumorigenesis, but decreases angiogenesis in the Matrigel plug assay. Whole transcriptome profiling of the landscape of MITF regulation in lung adenocarcinoma indicates that MITF is involved in cell development, cell cycle, inflammation and WNT signaling pathways. Chromatin immunoprecipitation assays revealed that MITF targets the promoters of *FZD7*, *PTGR1* and *ANXA1*. Moreover, silencing *FZD7* reduces the invasiveness that is promoted by silencing MITF. Strikingly, *MITF* has significantly inverse correlations with the expression of its downstream genes in lung adenocarcinoma. In summary, we demonstrate the suppressive role of MITF in lung cancer progression, which is opposite to the canonical oncogenic function of MITF in melanoma.

## INTRODUCTION

Lung cancer is the leading cause of cancer-related death worldwide [1]. Approximately 85% of lung cancers are non-small cell lung cancer (NSCLC) including adenocarcinoma, squamous cell carcinoma and large cell carcinoma, and lung adenocarcinoma accounts for the majority of all lung cancer cases [2]. The 5-year survival rate of early stage NSCLC ranges from 41% to 90%, whereas tumor metastasis or recurrence leads to the high mortality of stage I patients after surgery [2, 3]. In addition, lymph node metastasis is also a prognostic predictor for NSCLC survival [4]. Metastasis involves oncogenic cell transformation, cell motility and invasion and angiogenesis [5, 6]. Through functional genomics studies and an isogenic metastasis cell model in CL1-0, CL1-1 and CL1-5 lung adenocarcinoma cell lines, we discovered several metastasis-related genes [7–9]. However, the detailed processes and molecular mechanisms in lung cancer metastasis are still unknown. A comprehensive understanding of the role of these novel genes in lung metastasis is critical for the development of prognostic markers and therapeutic strategies.

MITF is a leucine zipper transcription factor with a basic helix-loop-helix (bHLH-Zip) structure and is essential for the differentiation and development of various cell types, including melanocytes, retinal pigment epithelium, osteoclasts and mast cells [10, 11]. Moreover, oncogenic MITF mediates melanoma progression in a “rheostat model” [12, 13]. In this model, high activity levels of MITF promote proliferation and differentiation, while low MITF levels increase invasion and metastasis and reduce proliferation [10, 13, 14]. However, a recent study indicated that melanoma cells with genetic ablation of SMAD7 exhibited a dual invasive/proliferative phenotype without suppression of MITF [15]. MITF mediates diverse melanoma phenotypes defined by distinct gene expression profiles and confers plasticity to melanoma cells [16]. Beyond being an activator, MITF can act as a repressor on the same gene promoter, such as ERBIN by recruiting FHL2 [17]. Thus, MITF is not only a survival lineage-specific transcription factor, but also regulates DNA damage repair, senescence, cell cycle, stemness, metabolism and invasion. To control the survival and invasiveness of melanoma, MITF activates the antiapoptotic gene *BCL2* and the cell cycle regulators, *CDK2* and *CDKN1A* [18–20], whereas MITF suppresses invadopodia formation by upregulating *GMPR* and *DIAPH1* expression, which influences the activities of small GTPases and cytoskeleton remodeling [13, 21]. Comprehensive transcriptome analysis reveals that MITF is involved in melanoma progression [22–24]. Although MITF has been extensively investigated in the progression of the melanocyte/melanoma linage, few studies have demonstrated the role of MITF in lung cancer.

In this study, we identified differential expression of *MITF* in our lung cancer metastasis cell model by expression microarrays. The clinical relevance of *MITF* expression for survival in NSCLC is analyzed in patients and public databases. We manipulated *MITF* expression in lung adenocarcinoma cell lines and measured cell invasion and migration activities. The influence of MITF on tumorigenesis and angiogenesis was evaluated in a xenograft mouse model. The landscape of the MITF-regulated transcriptome was profiled by expression microarrays, and the significant regulatory network of MITF was identified by pathway analysis. We further identified several targets of MITF in lung adenocarcinoma and clarified their effects on cancer progression by utilizing *in vitro* and *in silico* analyses.

## RESULTS

### *MITF* expression is associated with better outcome in NSCLC

**Figure 1:**
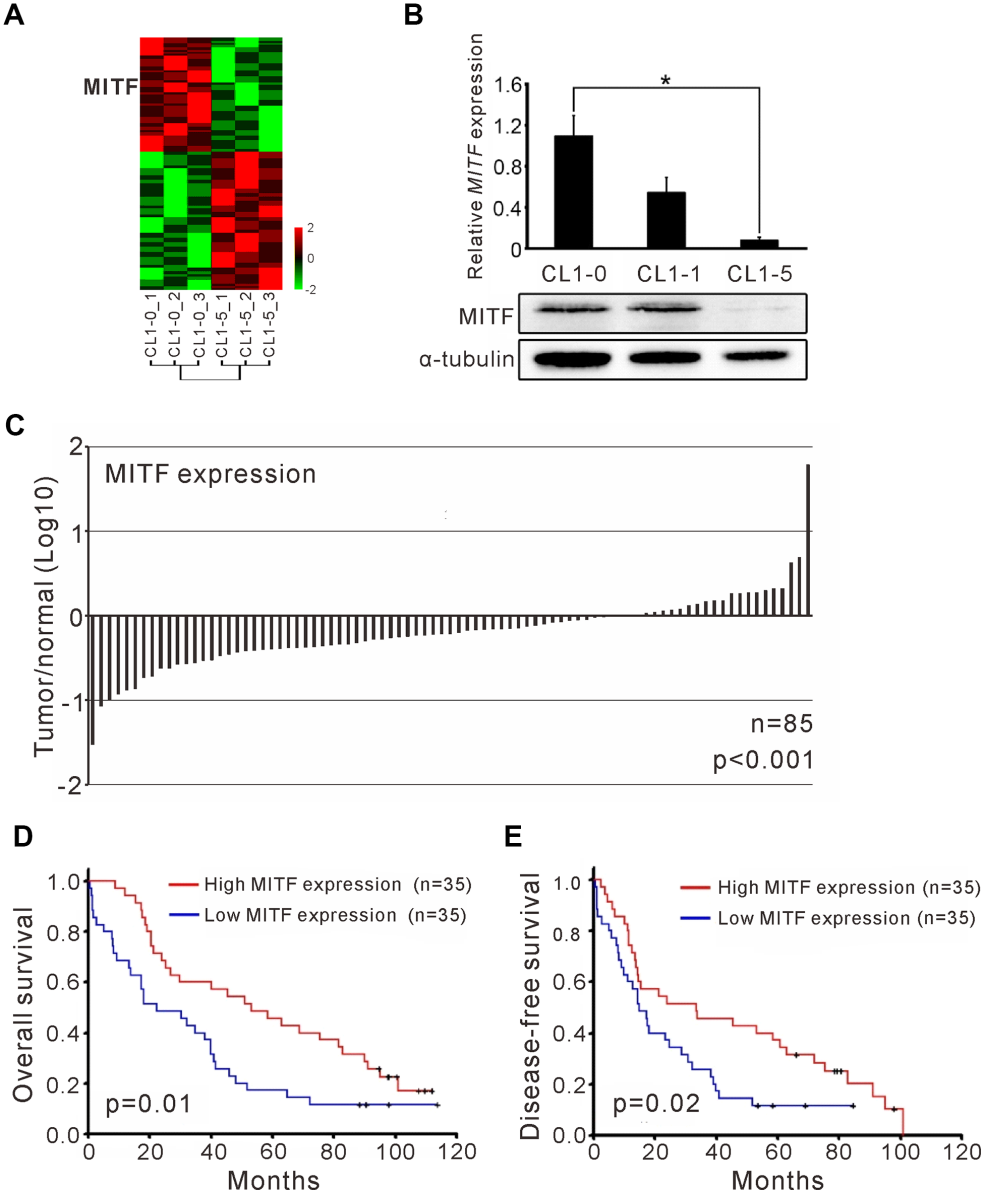
**The *MITF* expression associated with cancer invasiveness and better outcome of NSCLC patients.** (**A**) Heatmap of the gene expression profiles in CL1-0 and CL1-5 were presented. Each cell line was analyzed for three replicates and the significant microarray probes with 5-fold change were applied. MITF was one of the significant expressed genes. The scale was used the z-score. (**B**) *MITF* mRNA and protein level was measured by quantitative real-time PCR and immunoblot. *p < 0.05 (mean ± SD, n = 3) (**C**) The ratio of *MITF* expression in tumor and adjacent normal parts of NSCLC patients (n = 85). The scale is the base 10 logarithm of the ratio of *MITF* expression. The difference of *MITF* expression between the two groups was estimated by Wilscoxon matched-pairs test. (**D**) Kaplan–Meier survival analysis estimated the overall survival of NSCLC patients by the *MITF* expression. (**E**) Kaplan–Meier survival analysis estimated the disease-free survival of NSCLC patients by the *MITF* expression. The *MITF* expression of clinical specimen was measured by real-time RT-PCR with TaqMan probe. The p-value for survival was estimated by log-rank test.

In our previous studies, we utilized an expression microarray to profile the gene expression of isogenic lung adenocarcinoma cell lines with different invasive abilities (Figure 1A) [25]. In a comparison of the expression profiles of low-invasive CL1-0 cells and high-invasive CL1-5 cells, we found a gene, *MITF*, which had 7.5-fold lower expression in CL1-5 cells than in CL1-0 cells (Supplementary Figure 1A). *MITF* transcript expression was 25-fold higher in CL1-0 cells than in CL1-5 cells, as confirmed by RT-PCR, and the protein expression was markedly decreased in CL1-5 cells (Figure 1B). We found that *MITF* expression in adjacent normal tissues was significantly higher than that in NSCLC tumors (p < 0.001, Wilcoxon matched-pairs test) (Figure 1C). Next, we enrolled 70 NSCLC patients to evaluate *MITF* expression (Table 1). The patients with high *MITF* expression had significantly better overall survival (OS) and disease-free survival (DFS) than those with low *MITF* expression (OS, p = 0.01; DFS, p = 0.02, log-rank test) (Figure 1D, 1E). The *MITF* expression based dichotomy was not associated with age, sex, histology features, or clinicopathological stages (Supplementary Table 1). In univariate Cox regression analysis, the hazard ratio (HR) of *MITF* expression was significant (for OS, HR 0.51, 95% CI = 0.30 to 0.86, p < 0.05; for DFS, HR 0.54, 95% CI = 0.32 to 0.92, p < 0.05). In multivariate Cox regression analysis, the HR of *MITF* expression for OS remained significant (for OS, HR 0.52, 95% CI = 0.30 to 0.90, p < 0.05; for DFS, HR 0.61, 95% CI = 0.35 to 1.05, p =0.07) (Table 2). These data indicated that *MITF* was a prognostic marker for lung cancer progression. To clarify the role of *MITF* in other populations, we analyzed available microarray datasets by the KM-plotter [26]. The results showed that *MITF* expression was significantly associated with OS and progression-free survival (PFS) in lung adenocarcinoma (p-value 0.047 was for OS and 0.034 for PFS, log-rank test) (Supplementary Figure 1B, 1C). Our data suggested that *MITF* was an independent prognostic marker for NSCLC and might have a suppressive role in lung cancer progression.

**Table 1:** Clinicopathologic characteristic of NSCLC patients (n=70).

**Characteristic**	**Patients No. (%)**
**Age, mean (±SD)**	67.48±10.21
**Gender**	
Male	59 (84.29)
Female	11 (15.71)
**Stage**	
I	34 (48.57)
II	7 (10.00)
III	29 (41.43)
**Primary Tumor**	
T1 and T2	60 (85.71)
T3 and T4	10 (14.29)
**Regional Lymph Nodes**	
N0	38 (54.29)
N1, N2, and N3	32 (45.71)
**Cell Type**	
Squamous cell carcinoma	19 (27.14)
Adenocarcinoma	42 (60.00)
Large cell carcinoma	7 (10.00)
Mixed (small/large cell carcinoma)	1 (1.43)
Bronchioloalveolar carcinoma	1 (1.43)

**Table 2:** Univariate and multivariate Cox’s regression analysis of the overall survival and disease-free survival prediction factors.

**Univariate Cox’s regression**							
		Overall survival			Disease-free survival		
Variable		HR	95% CI	p-value	HR	95% CI	p-value
Age	<60	1			1		
	≥60	1.01	0.50-2.06	0.98	0.85	0.43-1.68	0.64
Gender	Male	1			1		
	Female	2.12	1.05-4.24	<0.05	1.54	0.77-3.08	0.22
Stage	I/II	1			1		
	III	3.36	1.97-5.74	<0.001	3.17	1.89-5.33	<0.001
Cell type	Adenocarcinoma	1			1		
	Others	1.18	0.70-1.99	0.54	1.20	0.71-2.03	0.50
MITF	High	1			1		
	Low	0.51	0.30-0.86	<0.05	0.54	0.32-0.92	<0.05
**Multivariate Cox’s regression**							
		Overall survival			Disease-free survival		
Variable		HR	95% CI	p-value	HR	95% CI	p-value
Age	<60	1			1		
	≥60	1.31	0.63-2.72	0.48	0.98	0.49-1.98	0.96
Gender	Male	1			1		
	Female	1.94	0.93-4.06	0.08	1.52	0.74-3.11	0.25
Stage	I/II	1			1		
	III	3.57	2.06-6.20	<0.001	3.07	1.81-5.23	<0.001
Cell type	Adenocarcinoma	1			1		
	Others	1.05	0.61-1.83	0.86	1.10	0.61-1.86	0.82
MITF	High	1			1		
	Low	0.52	0.30-0.90	<0.05	0.61	0.35-1.05	0.07

HR, hazard ratio; CI, confident interval of hazard ratio.

### MITF suppresses the cell migration and invasion in lung adenocarcinoma cells

**Figure 2:**
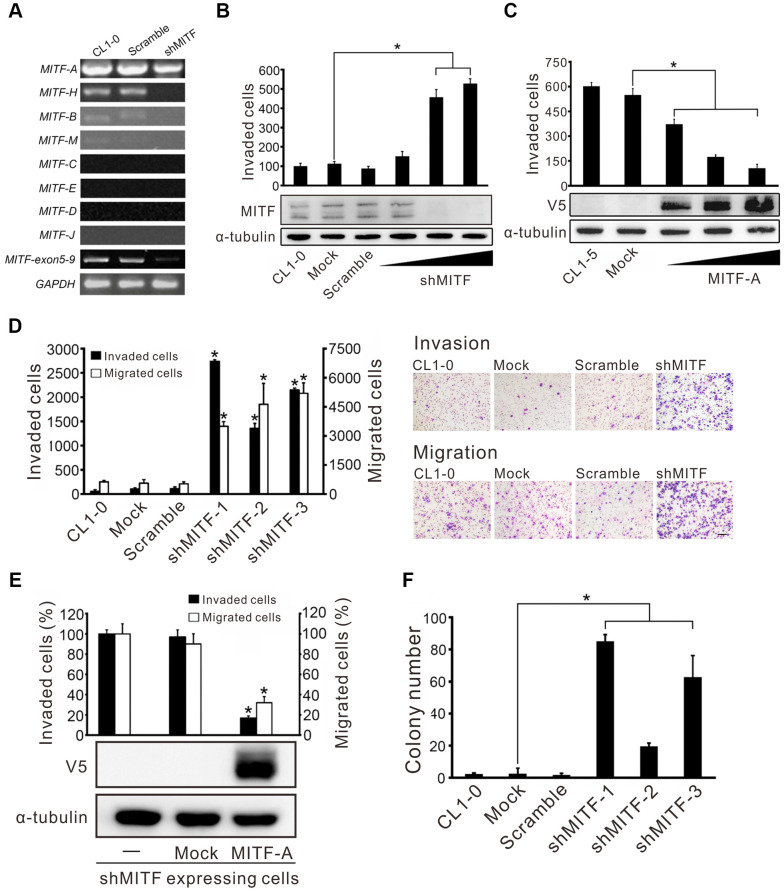
**MITF promotes cell invasion and migration in lung adenocarcinoma cells.** (**A**) The silencing efficiency of shMITF to different MITF isoforms in CL1-0 cells was measured by isoform-specific RT-PCR. Controls included RT-PCR for the common MITF exons 5-9 and GAPDH. (**B**) The cell invasive ability of CL1-0 cells was assayed after transiently delivering the shMITF expressing plasmids with different doses. Mock: vector transfectant; Scramble: scramble transfectant. *p < 0.05 (mean ± SD, n = 3) (**C**) The cell invasive ability of CL1-5 cells was assayed after overexpression of MITF-A with different doses. *p < 0.05 (mean ± SD, n = 3) (**D**) The cell invasive and migratory abilities of stably *MITF*-silenced cells were assayed by using Boyden chamber assays with and without Matrigel, respectively. *p<0.05 (mean ± SD, n = 3) (**E**) Re-expressed MITF-A in stably *MITF*-silenced cells were assayed for the cell invasion and migration. *p < 0.05 (mean ± SD, n = 3) (**F**) The anchorage-independent colony formation ability of stably *MITF*-silenced cells was assayed. Scale bar, 100 μm. *p < 0.05 (mean ± SD, n = 3).

Given the existent transcripts of various MITF isoforms, the expression of different MITF isoforms in CL1-0 lung adenocarcinoma cells was measured. We found that *MITF-A* was the most abundant isoform in lung cancer cells, and *MITF-H*, *MITF-B* and *MITF-M* were the lesser abundant isoforms (Figure 2A). Next, the impact of MITF on invasiveness was evaluated. shMITF was transiently expressed in low-invasive CL1-0 cells and the number of invaded cells was increased in a dose-dependent manner (Figure 2B). The multiple bands detected by high-resolution Western blotting demonstrated the isoform expression of *MITF*. Overexpression of *MITF-A* in highly invasive CL1-5 lung adenocarcinoma cells reduced cell invasive ability (Figure 2C). Next, we selected three clones stably expressing shMITF by antibiotics to avoid clonal bias (Supplementary Figure 2A). The three *MITF*-knockdown clones showed an increase in cell migration and invasion (Figure 2D). Introducing the *MITF-A* expressing plasmid into *MITF*-silenced cells reduced the migratory and invasive abilities by 60% and 80%, respectively (Figure 2E). *MITF* silencing promoted the anchorage-independent colony growth of CL1-0 cells (Figure 2F). However, MITF did not appear to affect cell proliferation (Supplementary Figure 2B). Taken together, the results indicated that *MITF* suppressed both cell migration and invasion in lung adenocarcinoma cells.

### Silencing MITF promotes tumorigenesis and metastasis but suppresses angiogenesis

**Figure 3:**
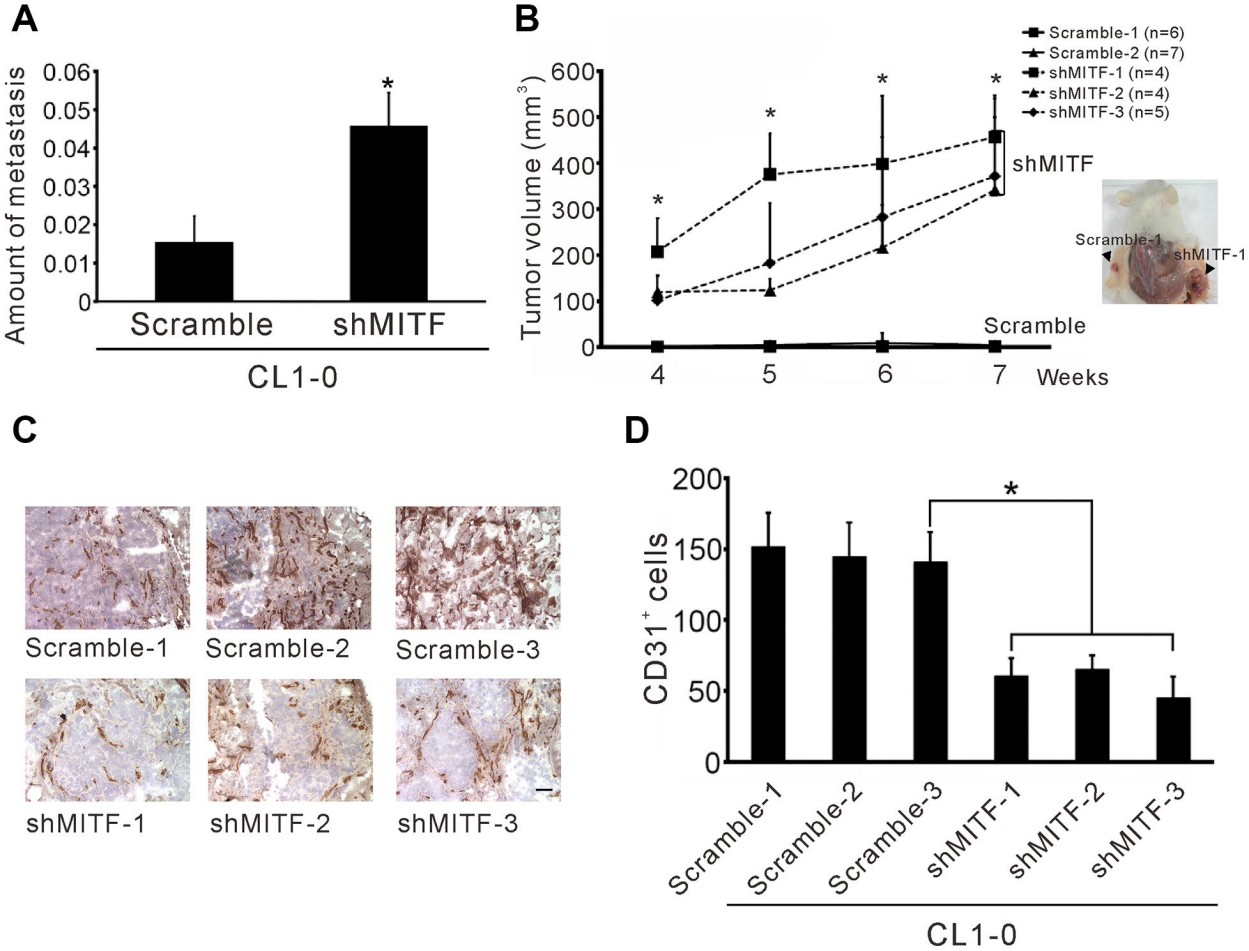
**Knockdown of *MITF* increases metastasis and tumorigenesis but inhibits angiogenesis.** (**A**) Intravenous injection with stable shMITF-harboring and scramble CL1-0 cells to SCID mice. The lung metastases were estimated by using real-time PCR for detection of the human Alu repeats. (**B**) Subcutaneous injection with stable shMITF-harboring cells into the right dorsal region and scramble cells into the left dorsal region of SCID mice. The tumor volume was measured every week. The mouse number of each group is listed on the plot. *p < 0.05 was calculated by ANOVA (mean ± SD) (**C**) The cells mixed with Matrigel and injected subcutaneously. At 10 days, the plug was dissected and assayed the CD31 positive cells by immunochemistry. Scale Bar, 25 μm. (**D**) The CD31 positive cells were counted by two blinded observers. A total of 21 fields/group (3 plugs × 7 fields) were analyzed. *p<0.05 (mean ± SD).

To investigate whether MITF plays a critical role in lung metastasis *in vivo*, we modified the method developed by Zijlstra et al. [27]. The results showed that *MITF*-knockdown in CL1-0 cells significantly increased lung metastasis compared to the scramble control (Figure 3A). Moreover, we also found that reduced *MITF* expression strongly enhanced tumorigenesis in SCID mice (p < 0.001) (Figure 3B). New capillary formation was evaluated by the Matrigel plug assay *in vivo*. Interestingly, a more than 60% reduction in the number of CD31-positive stained endothelial cells in the *MITF*-knockdown tumors was observed at 10 days after injection (Figure 3C, 3D). Overall, *MITF* suppressed tumorigenesis and distal metastasis, but promoted angiogenesis *in vivo*.

### MITF targets *ANXA1*, *FZD7* and *PTGR1*, and suppresses cell invasion by regulating *FZD7*

**Figure 4:**
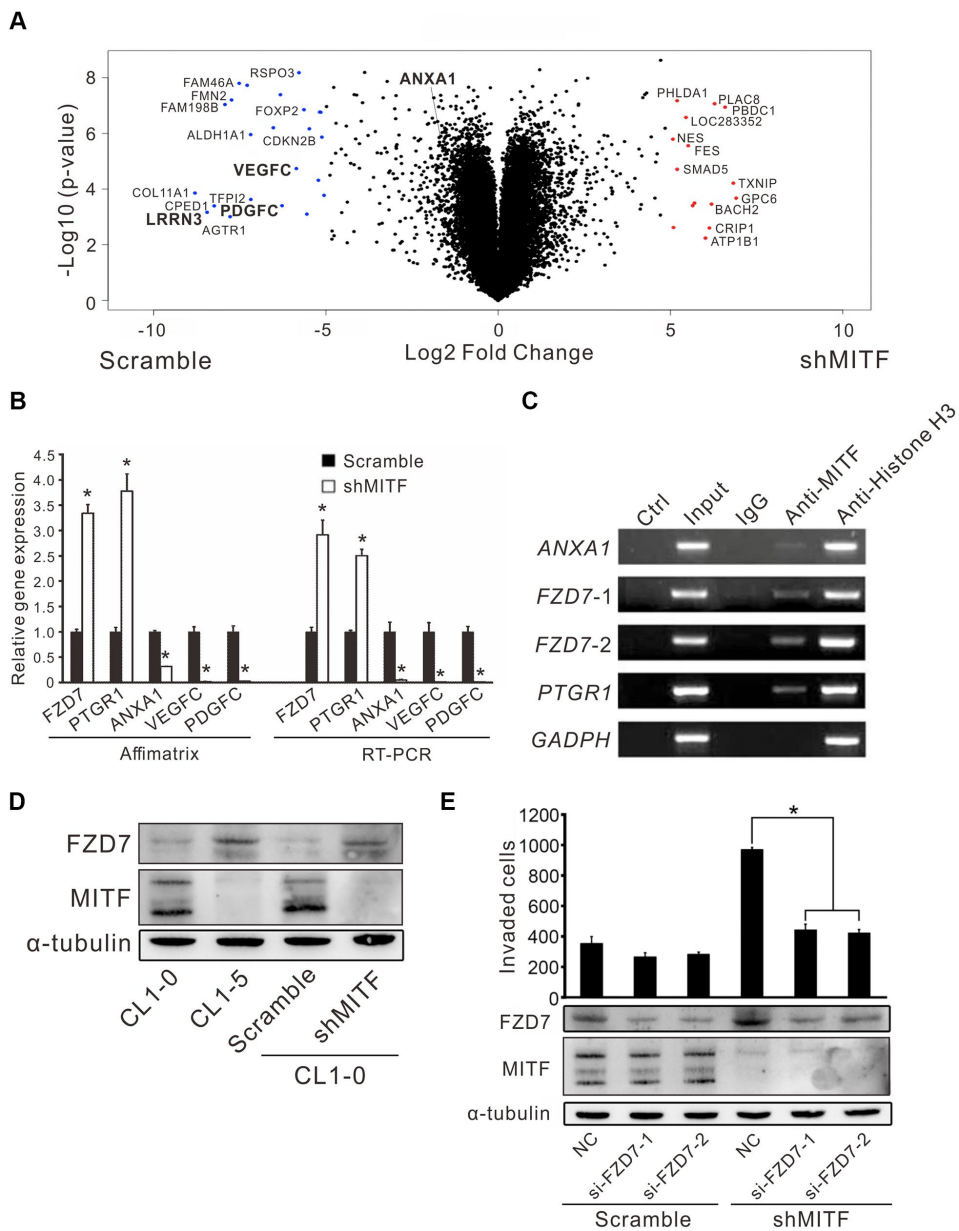
**The regulation landscape and the direct targets of MITF.** (**A**) The volcano plot showed the significant MITF-altered genes with log2-fold-change. Red dot: genes with log2-fold-change ≥ 5, p < 0.05; Blue dot: genes with log2-fold-change ≤−5, p < 0.05. (**B**) Relative gene expressions in CL1-0 scramble cells and stably MITF-silenced cells assayed by expression microarray and real-time RT-PCR. The scramble and three stably MITF-silenced clones were assayed in triplicate. *p < 0.05 (mean ± SD, n = 9). (**C**) MITF binding targets identified by chromatin immunoprecipitation assay. (**D**) MITF negatively regulated FZD7 expression assayed by immunoblot. (**E**) The cell invasion of CL1-0 scramble cells and stably MITF-silenced cells assayed after silencing *FZD7*. *p < 0.05 (mean ± SD, n = 3).

MITF is an important transcription factor in cell differentiation and cancer progression, and its transcriptional targets have been investigated in previous studies [12–14, 16, 17]. However, the landscape of MITF targets in NSCLC is not fully understood. Thus, we profiled the whole transcriptome of shMITF stably expressing cells and scramble cells by expression microarrays (Figure 4A). A total of 1,190 differentially expressed genes with greater than 2-fold changes were identified and applied for gene set enrichment analysis (GSEA) and MetaCore version 19.4 analysis [28, 29]. The resulting pathways were predominantly related to inflammation, development, cell signaling and cell cycle (Supplementary Tables 2, 3). We focused on several significant genes that were predicted targets of MITF and involved in those pathways. Real-time PCR and chromatin immunoprecipitation assays were performed to evaluate whether MITF regulated them directly. Compared to the scramble control, in *MITF-*silenced cells the frizzled homolog 7 (*FZD7*) and leukotriene B4 12-hydroxydehydrogenase *(PTGR1*) expression increased 3-fold and 5-fold, respectively, whereas annexin A1 (*ANXA1*) expression was suppressed approximately 25-fold (Figure 4B). Additionally, *VEGFC* and *PDGFC* in MITF-silenced cells decreased to approximately 0.02 of the expression in scramble cells, which was consistent with the *in vivo* angiogenesis results. Moreover, MITF was able to bind to the promoters of these genes, *ANXA1*, *FZD7* and *PTGR1* (Figure 4C). Additionally, the WNT signaling pathway was of interest because of its high ranking in the pathway analysis (Supplementary Tables 2, 3). Since FZD7 is a transmembrane receptor in the WNT pathway, we further investigated whether MITF regulated cell invasion through FZD7 in lung cancer cells. In Figure 4D, silencing of *MITF* resulted in an increase in FZD7 protein. Furthermore, knockdown of *FZD7* with two independent siRNAs significantly inhibited shMITF-induced invasive ability (Figure 4E). These data suggested that MITF transcriptionally regulated *ANXA1*, *FZD7* and *PTGR1* and regulated cell invasion through FZD7.

### Inverse correlations of *MITF* and *ANXA1* expression in lung adenocarcinoma and melanoma

**Figure 5:**
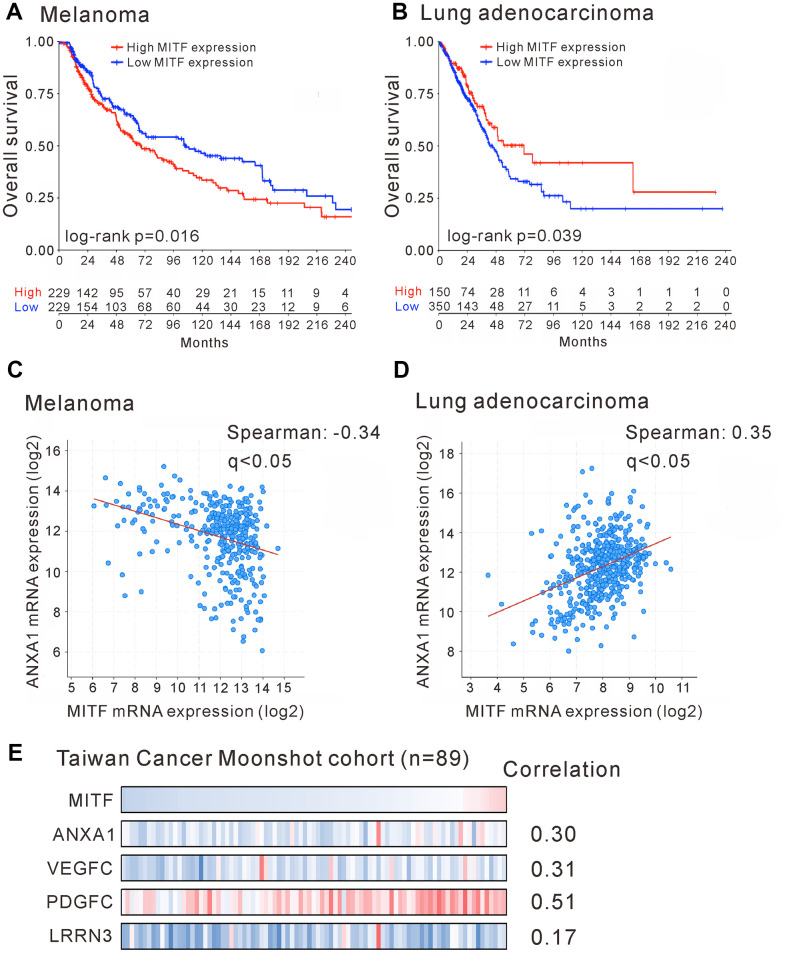
**Reversal association and correlation of *MITF* in clinical significance.** Kaplan-Meier survival estimated the association of *MITF* expression and survival of patients with skin cutaneous melanoma (SKCM) (**A**) or with lung adenocarcinoma (LUAD) (**B**) in TCGA database. The correlation of *MITF* and *ANXA1* expression in skin cutaneous melanoma (n = 363) (**C**) or in lung adenocarcinoma (n = 507) (**D**). The TCGA PanCancer Atlas database was analyzed by cBioPortal. (**E**) The mRNA log2 T/N ratio of indicated genes of 89 lung adenocarcinoma patients from Taiwanese cohort and the Spearman’s correlation with *MITF*. q < 0.05 was considered statistically significant.

To investigate the significance of *MITF* in malignancies, we analyzed The Cancer Genome Atlas (TCGA) database by OncoLnc and the pathology atlas [30, 31]. *MITF* is a well-known protumorigenic gene expressed in melanocytes. Indeed, *MITF* expression was significantly associated with poor outcome in skin cutaneous melanoma (SKCM) (p < 0.05, log-rank test) (Figure 5A and Supplementary Table 4). In contrast to melanoma patients, lung adenocarcinoma (LUAD) patients with high *MITF* expression had longer survival time than those with low *MITF* expression (p < 0.05, log-rank test) (Figure 5B and Supplementary Table 5). The results demonstrated an inverse association of *MITF* in lung adenocarcinoma.

The distinct functions of MITF in lung adenocarcinoma and melanoma attracted our interest. We hypothesized that the opposite transcriptional regulation of MITF resulted in different effects on these two malignancies. We evaluated the expression correlation of MITF and its targets between lung adenocarcinoma and melanoma by TCGA PanCancer Atlas database with cBioPortal analysis [32, 33]. We first found that ANXA1 expression was positively correlated with MITF expression in lung adenocarcinoma (Spearman’s correlation = 0.35, q < 0.001) but negatively correlated with MITF in melanoma (Spearman’s correlation = −0.34, q < 0.001) (Figure 5C, 5D). Furthermore, we searched for genes that had a significant correlation with MITF expression in lung adenocarcinoma or melanoma. A total of 2,160 and 2,186 genes had significant correlations with MITF in LUAD and SKCM databases, respectively (Spearman’s correlation > 0.3 or < −0.3, q-value < 0.05) (Supplementary Figure 3). After intersecting and analyzing both gene sets, we further found that most of the genes with a positive correlation with MITF expression in lung adenocarcinoma had a negative correlation with MITF in melanoma (354 genes out of 446 total genes; p = 1.01E−10, Chi-square test) (Table 3). Conversely, there were 17 genes with a reverse correlation. Among these 371 genes, we found that VEGFC, PDGFC and LRRN3 were extremely downregulated in MITF-silenced lung adenocarcinoma cells (log2-fold-change < −5, p < 0.05) (Figure 4A and Supplementary Table 6). Consistent with the TCGA database, MITF was positively correlated with ANXA1, VEGFC, PDGFC and LRRN3 in the East Asian cohort (Figure 5E) [34]. Furthermore, the expression levels of ANXA1, VEGFC, PDGFC and LRRN3 were significantly positively associated with the survival of lung adenocarcinoma (Supplementary Figure 4A–4D). The results suggested that MITF has opposite functions in lung adenocarcinoma and melanoma, possibly by positively or negatively regulating its downstream targets.

**Table 3:** The number of genes with significantly positive or negative correlation with *MITF* expression in lung adenocarcinoma and melanoma.

**Lung Adenocarcinoma**					
		**Positive**	**Negative**	**Total**	*p*-value*
**Melanoma**	Positive	67	17	84	1.01E-10
	Negative	354	8	362	
	Total	421	25	446	

The significant correlation were determined by q-value <0.05.

Positive: Spearman's correlation >0.3; Negative: Spearman's correlation <−0.3.

*Chi-square test was used for the statistical analyses.

## DISCUSSION

MITF is a critical transcription factor that regulates the cell cycle, cell differentiation and cancer progression. The oncogenic role of MITF in melanoma is well defined, but the function of MITF in the NSCLC is still unclear. *MITF* contains several isoforms generated through differential use of alternative promoters that are controlled by tissue specificity, leading to MITF isoforms having distinct N-termini with a range of 419-526 amino acids [10]. MITF-A is the largest protein isoform and regulates kidney development [35]. Except for MITF-M, all isoforms contain exon 1B1b, which facilitates MITF interaction with RAG GTPases at the lysosomal surface and controls MITF nuclear-cytosol transportation [36, 37]. The expression of the shortest isoform *MITF-M* is limited to melanocytes and melanoma cells, but *MITF-A* is the major isoform expressed in our low-invasive lung adenocarcinoma cell line. *MITF-A* suppressed the invasive ability of CL1-5 cells in a dose-dependent manner that was distinct from the action of *MITF-M* in melanoma cells (Figure 2C).

Chromatin immunoprecipitation (ChIP) sequence analysis of melanoma cells indicates that MITF potentially binds between 12,000 and 100,000 genomic sites and that ~9400 of those lie within 20 kb of an annotated RefSeq gene, but genes may not obviously exhibit MITF regulation even though they have high levels of MITF occupancy at their promoter [22, 24]. We found that *ANXA1*, *FZD7* and *PTGR1* were MITF direct targets in CL1-0 cells, but these genes were not regulated in melanoma 501MEL cells [22]. However, the genes *ANXA1*, *PDGFC, VEGFC* and *LRRN3* were downregulated in CL1-0 shMITF-harboring cells, while they were upregulated in si-MITF melanoma 501MEL cells. In addition, they positively correlated with *MITF* in the LUAD TCGA database, but negatively correlated in the SKCM TCGA database. Accumulated data suggest that MITF executes tumor suppressive or oncogenic functions by switching its transcriptional role and targets in different malignancies.

ANXA1 is regarded as a proinvasive protein in melanoma and is correlated with poor outcome of lung cancer [38, 39], but it is associated with longer survival of NSCLC nonsmoking female and pancreatic ductal adenocarcinoma patients [40, 41]. Knockdown of *ANXA1* in pancreatic ductal adenocarcinoma increases cell migration and invasion, but inhibits cell proliferation, which is similar to the phenotype at low levels of MITF in melanoma [41]. Although reduction of *ANXA1* in H1299 and A549 cells suppresses cell proliferation and invasion [42], in most cases, ANXA1 acts as a tumor suppressor inhibiting tumor growth [43]. Due to its numerous, diverse, and sometimes opposing functions, ANXA1 has been described as a “double-face” protein [44]. PTGR1 is known as leukotriene B4 12-hydroxydehydrogenase (LTB4DH), which is capable of inhibiting lung cancer growth in nude mice and inactivating prostaglandins and the leukocyte chemoattractant leukotriene B4 (LTB4) [45, 46]. The tumor microenvironment of melanoma is shaped by the level of MITF expression and depletion of MITF stimulates the release of inflammatory cytokines such as IL-6 and IL-1β [47, 48]. We showed that MITF binds to the promoter of *PTGR1* and downregulates *PTGR1* expression in lung adenocarcinoma. The fundamental function of PTGR1 in lung cancer progression is unclear but MITF-mediated inflammation involved in regulating *PTGR1* has been proposed.

β-Catenin activates *MITF* expression and acts as the coactivator for MITF to drive downstream gene expression [49, 50]. Wnt3a stabilizes the MITF protein and MITF enhances WNT signaling by driving lysosome biogenesis [51]. We found that MITF is involved in the canonical WNT pathway and binds to the promoter of *FZD7*. Supposedly, MITF enables transcriptional repression of *FZD7*, which encodes a receptor accounting for signaling canonical WNT pathways [52]. GSEA indicated that WNT signaling decreased in *MITF*-silenced cells, but depletion of *MITF* compromised the increase in *FZD7* expression. Downregulated FZD7 rearranges the actin cytoskeleton and strengthens cell–cell adhesion by inhibiting RhoA and activating Rac1 [53]. In agreement with that, *FZD7* is upregulated in CL1-0 shMITF cells and contributes to shMITF-induced invasiveness (Figure 4E). In proliferative melanoma cells, many MITF and WNT target genes are upregulated, but in the invasive melanoma cells, they are simultaneously downregulated [54]. After depletion of *MITF* expression in lung adenocarcinoma, cell proliferation was unaltered but tumorigenesis and metastases increased, although cell cycle and proliferation were enriched pathways. The rheostat model of MITF in lung adenocarcinoma is paradoxical, but the reciprocal regulation between MITF and WNT signaling matters.

The role of MITF in angiogenesis has been less investigated in lung adenocarcinoma. We found that the expression of the angiogenic factors *VEGFC* and *PDGFC* decreased over 60-fold in shMITF-harboring cells compared with control cells. This result corresponds to less endothelial cell formation in shMITF tumors. In contrast to their expression in lung adenocarcinoma, *VEGFC* and *PFGFC* are upregulated by silencing *MITF* in melanoma cells, which is in accordance with MITF low-expression promoting metastasis because angiogenesis usually accelerates metastasis [22]. However, the induction of vessel maturation and normalization accounts for the inhibition of both tumor growth and metastasis [55]. In cardiomyocytes, upregulated MITF increases VEGF production and promotes angiogenesis [56]. Moreover, the expression of *VEGFC* and *PDGFC* was associated with longer survival time of lung adenocarcinoma patients. Accordingly, MITF-mediated increase in angiogenesis in lung adenocarcinoma may be relevant to inhibition of both tumor growth and metastasis.

**Figure 6:**
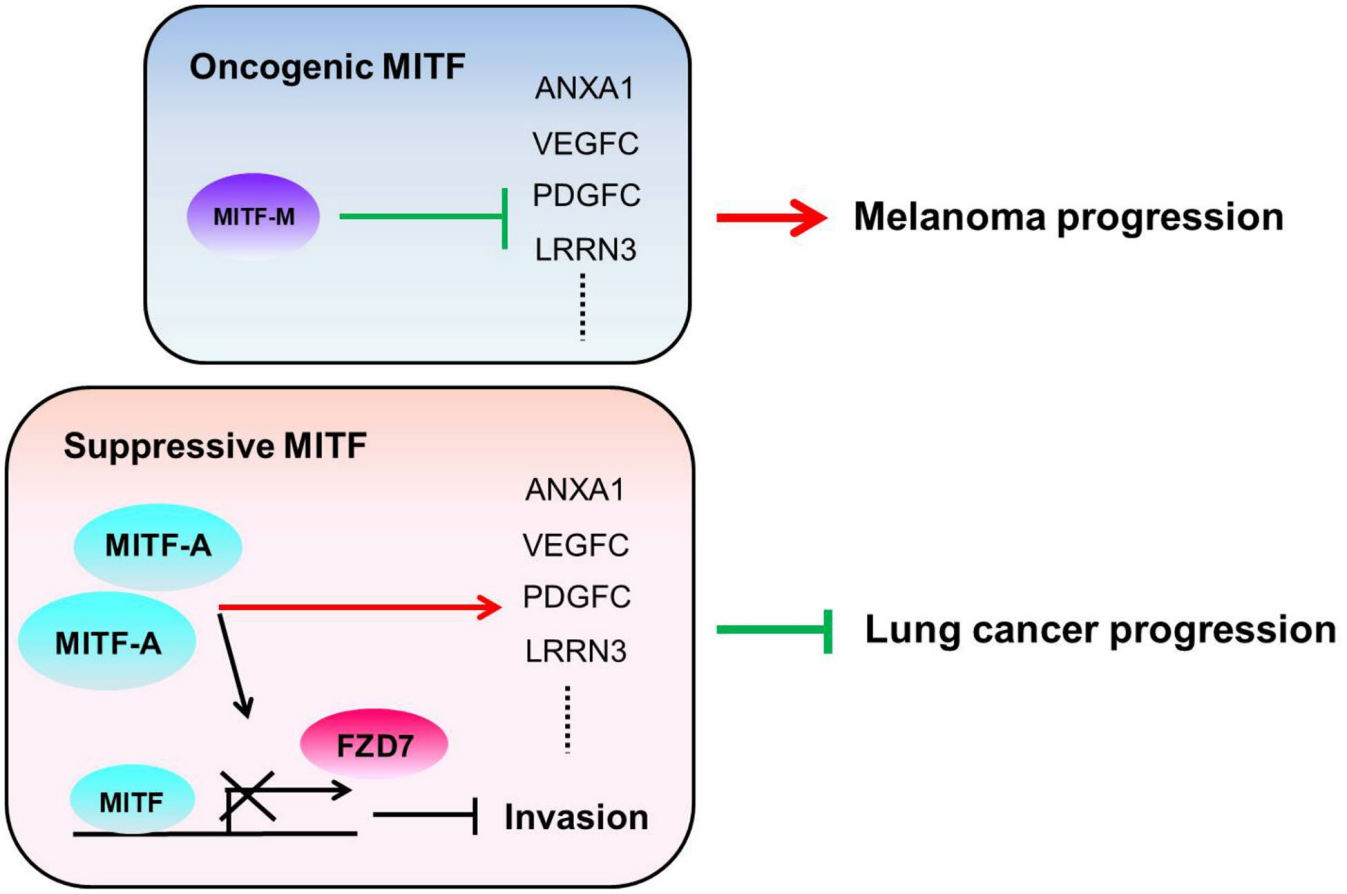
**The illustration of the role of MITF in lung adenocarcinoma and melanoma.** We hypothesize that the different MITF isoforms and their transcriptional regulations lead to opposite impacts on cancer progression. The dominant isoform *MITF-A* expresses in CL1-0 cells and inhibits cell invasion by repressing FZD7 expression. Additionally, MITF activates *ANXA1*, *VEGFC*, *PDGFC* and *LRRN3* in lung adenocarcinoma, but suppresses *ANXA1*, *VEGFC*, *PDGFC* and *LRRN3* in melanoma. The red lines indicate the “activate” and the green lines indicate the “repress”.

The transcriptional landscape of MITF regulation in lung adenocarcinoma was evaluated and was associated with inflammation, development, cell cycle and WNT signaling pathways, which is consistent with previous studies [10, 57]. However, we found that MITF plays a suppressive role in lung cancer progression and is a favorable prognostic marker for overall survival in NSCLC, which is contrary to the role of MITF in melanoma. In our study, we focused on MITF-mediated metastasis. However, while some lung cancer patients responded to chemo-/radiotherapy, there was almost no response to chemo-/radiotherapy or any therapy in metastatic melanoma patients until vemurafenib received approval for melanoma treatment [58]. Therefore, the prognosis of lung cancer patients might be determined by their responses to chemo-/radiotherapy and might be associated with stem-like phenotype/MITF-low expression [59], whereas melanoma patients’ prognosis might be determined largely by tumor growth and might be associated with differentiated state/MITF-high expression. Whether MITF regulates stemness and is associated with chemoresistance of lung cancer cells is an unanswered question. By analyzing both malignancies by microarrays and clinical datasets, we found an inverse pattern of gene expression and correlations with *MITF*. Although not all significantly MITF-correlated genes in clinical databases are transcriptional targets of MITF, they may be involved in the MITF regulation in some contexts. Approximately 83% of the genes that significantly correlate with *MITF* expression in the LUAD database have reverse correlations in SKCM (371 genes in total 446 genes) (Table 3). This finding is in agreement with genes with opposite expression in CL1-0 cells and 501MEL cells. Among these genes, *ANXA1* is one of the MITF transcriptional targets. As expected, ANXA1 plays multiple roles in cancer progression similar to MITF. We hypothesize that the transition between being an activator or a repressor and the selective regulation of downstream targets enable MITF to differentially regulate diverse functions in different malignancies, and ANXA1 accounts at least in part for the diverse effects of MITF (Figure 6). Which function mediated by ANXA1 in the context of MITF regulation in lung adenocarcinoma and melanoma progression, and how MITF regulates ANXA1 warrant further investigation.

Collectively, our findings provide a new insight into the role of MITF (Figure 6). Knockdown of *MITF* increased cell invasiveness, tumorigenesis and metastasis, but decreased angiogenesis. *MITF* expression was significantly associated with favorable OS and DFS in NSCLC. Depletion of *MITF* enhanced cell invasion and migration by increasing *FZD7*, while knockdown of *FZD7* reduced shMITF-induced invasiveness. *MITF* had a positive correlation with its target *ANXA1* in lung adenocarcinoma, but had an inverse correlation in melanoma. Our data suggest that *MITF* plays a suppressive role in lung cancer progression and serves as a prognostic marker of NSCLC. Further investigation is needed to elucidate the “rheostat model” of MITF in lung adenocarcinoma, and which transcriptional partners or modifications for MITF to shift its role in the progression of different malignancies.

## MATERIALS AND METHODS

### Patients and tissue specimens

A total of 70 treatment-naïve patients who underwent surgical resection and with histologically confirmed NSCLC were obtained from the Taichung Veterans General Hospital from November 1999 to August 2002. None of the patients had received neoadjuvant chemotherapy or radiation therapy. The clinicopathologic characteristic of them was shown in Table 1. Adjacent normal and tumor paired specimens of 85 NSCLC were obtained from another cohort. Informed consents were obtained from all patients and this investigation was approved by the Institutional Review Board of the Taichung Veterans General Hospital. All lung cancer patients were staged according to the American Joint Committee on Cancer Staging (AJCC) and the histology was performed with World Health Organization standards.

### Cell culture and transfection

The human lung adenocarcinoma cell lines, CL1-0, CL1-1, and CL1-5 in ascending order of invasive competence were kind gifts from Professor Pan-Chyr Yang (National Taiwan University, Taipei, Taiwan) and were established in previously studies [60]. Cells were cultured in RPMI-1640 medium (Gibco, Life technologies, Carlsbad, CA) with 10% fetal bovine serum. Short hairpin RNA (shRNA) used in MITF (GenBank NM_000248) silencing studies was purchased from Open Biosystems (Huntsville, AL). [61] The shRNA sequence targeting the human MITF gene is 5’-GCTAAAGTGATAGAAAGGCACCGCCTTACCCAAGTAAAGCAGTACCTTTCTACCACTTTAGC-3’ (the underline sequence matches MITF open reading frame nucleotide 94-122). A scrambled shRNA (5’-TGCTGTTGACAGTGAGCGATCTCGCTTGGGCGAGAGTAAGTAGTGAAGCCACAGATGTACTTACTCTCGCCCAAGCGAGAGTGCCTACTGCCTCGGA-3’) which does not match any known mammalian gene was used as the scramble control. CL1-0 cells were transfected with shRNAs using the Lipofectamine^TM^ 2000 (Life technologies, Carlsbad, CA) and selected with 2.5 μg/ml puromycin (Sigma, St Louis, MO) for stably MITF-silenced transfectants according to manufacturer’s instructions. The full-length human *MITF-A* cDNA (GenBank NM_198159) was amplified from CL1-0 cells by RT-PCR and primers (forward primer: GCCATGCAGTCCGAATCGGG and reverse primer: ACAAGTGTGCTCCGTCTCTTCCA) and cloned into the constitutive mammalian expression vector pEF6/V5-His TOPO (Life technologies, Carlsbad, CA).

### Real-time RT-PCR

The mRNA expression level of MITF was detected by qRT-PCR on ABI prism 7900 sequence detection system (Applied Biosystems, Branchburg, NJ), performed in accordance with the manufacturer instructions. For the SYBR Green method, the MITF primers used were the following: forward primer MITF-F: 5’-CCGGCATTTGTTGCTCAGA-3’ and reverse primer MITF-R: 5’- AGACCCGTGGATGGAATAAGG-3’ as well as the TATA box-binding protein (TBP) TBP-F: 5’-TTTTCTTGCTGCCAGTCTGGAC-3’ and TBP-R: 5’-CACGAACCACGGCACTGA TT-3’. TBP was used as the internal control. For the TaqMan method, the sequences of customized MITF detection probes were as follows: MITF forward primer: 5’-CCGGCATTTGTTGCTCAGA-3’, reverse primer: 5’-ACTTGAAATGCAGGCTC-3’, and the probe sequence: 5’- ACTTGAAATGCAGGCTC-3’. The TBP detection probe (Assay ID: Hs00427621_m1, Applied Biosystems, Branchburg, NJ) was used as the internal control. Other primers for MITF target genes were listed in Supplementary Table 7. All assays were performed in triplicate of three independent experiments.

### Migration, invasion assays and colony formation

Transwell culture inserts (Costar, Cambridge, MA) were used for the assessment of cell migration and extracellular matrix invasion [8]. In migration assay, 1 × 105 cells in 200 μl of serum-free RPMI were seeded on top of transwell and incubated for 8 hours. For invasion assay, the filter was coated with a thin layer of Matrigel matrix (R&D System, Minneapolis, MN). The 1 × 105 cells were seeded onto the coated matrix and incubated for 18 hours. The number of migrated and invaded cells were counted at 200 × magnification under a light microscope. To determine anchorage independent colony formation assay, six-well plates were first layered with 1ml 0.7% low-melting point agarose in PBS. In the second layer, 100 cells per well were suspended in 1 ml RPMI containing 0.35% low-melting point agarose. 1ml RPMI was covered on the second layer. The plates were incubated for 4 weeks and then washed by PBS, fixed in 4% paraformaldehyde, and stained with 0.5% crystal violet. Colonies with a dimeter greater than 1mm were counted under an inverted microscope.

### Experimental metastasis assay *in vivo*

A single-cell suspension containing 1 × 106 cells in 100 μl of PBS was injected into lateral tail vein of 6-week-old SCID mice. Mice were sacrificed after 10 weeks. The detection of human tumor cells in mouse lung was based on the human Alu element presented in mouse lung DNA extracts, which is modified from the method developed by Zijlstra et al. [27]. Genomic DNA was extracted from harvested tissues using the genomic DNA purification kit (Qiagen, Hilden, Germany). Primers specific for the human Alu element (reverse: 5-CGCCTGTAATCCCAGCTACT-3 and forward: 5-GATCTGGCTCACTGCAAC-3) and universal ProbeLibrary probe: #2 (Roche Diagnostics, Alameda, CA) were used to detect the human Alu repeats present in genomic DNA from mouse tissues. Each PCR contained 30 ng of genomic DNA was performed in a final volume of 20 μl with the ABI prism 7900 system (Applied Biosystems, Branchburg, NJ). Mouse DNA was detection of with mGAPDH primers (reverse: 5-AGCTTGTCATCAACGGGAAG-3 and forward: 5-TTTGATGTTAGTGGGGTCTCG-3) and Universal ProbeLibrary probe: #9. The relative quantity of Alu against that of mouse GAPDH was defined as −ΔCT = −[CTAlu − CTGAPDH]. Relative changes in metastasis were then calculated as 2 –ΔCT × K, where K is a constant and the experimental samples in triplicate.

### Angiogenesis and tumorigenesis *in vivo*

The 4 × 106 cells in 200 μl PBS were subcutaneously injected into the dorsal region of SCID mice. Injected mice were examined every week for tumor appearance and tumor volumes were estimated from the length (a) and width (b) of the tumors using the formula V = ab2/2. The 5 × 105 cells were mixed with 200 μl growth factor reduced Matrigel (Becton Dickinson, Bedford, MA) and injected into the dorsal region of SCID mice. After 10 days, the Matrigel plug was dissected out, frozen, and fixed with acetone. Sections of the Matrigel plug (5 mm) were stained with an endothelium-specific anti-mouse CD31 monoclonal antibody (Abcam, Cambridge, England) for infiltrated endothelial cells. The CD31 positive cells were counted by blinded observers on a 200 × microscopic field. A total of 21 fields/group (3 plugs × 7 fields) were analyzed.

### Microarray analysis

cRNA preparation and array hybridization were performed according to the Affymetrix GeneChip Expression Analysis Technical Manual. The biotinylated RNA was fragmented and hybridized overnight to Human genome U133 plus 2.0 GeneChip (Affymetrix, Santa Clara, CA). The raw data were processed using GC-RMA algorithm. All hybridization experiments were performed in biological triplicate with cRNA probes prepared from three different MITF shRNA transfectants (shMITF-1, shMITF-2, shMITF-3) and three different scramble ones (scramble-1, scramble-2 and scramble-3). These array data had been uploaded into GEO with GSE146868.

### Isoform-specific RT-PCR

Total RNA was isolated by the TRIzol reagent (Life Technologies, Carlsbad, CA). and reverse-transcribed using SuperScript^TM^ II (Life Technologies, Carlsbad, CA) and random primers. Subsequently, 15 ng of the cDNA was used to analyze the presence of each isoform using PCR amplification. Isoform-specific 5’ primers were used for each reaction with a common 3’ primer (MITF-com R) in exon 5 (Supplementary Table 7) [62]. To detect total MITF expression, primers were designed to amplify a conserved fragment from exon 5 (forward: MITF-exon5 F) to exon 9 (reverse: MITF-exon9 R) and GAPDH was acted as an internal control. Cycling times and temperatures for PCR were 94° C for 30 sec, 55° C for 30 sec, and 72° C for 1 min for 35 cycles except 30 cycles for MITF exon 5-9. PCR products were resolved on a 2% agarose gel.

### Chromatin immunoprecipitation assay and immunoblot

Transcription start sites of selected MITF-regulated genes were obtained from the Transcriptional Regulatory Element Database (http://rulai.cshl.edu/cgi-bin/TRED/). TRANSFAC Professional database (Biobase Biological Databases) was used to identify potential MITF binding sites for primer designed (Supplementary Table 8). The chromatin immunoprecipitation assay was performed according to the protocol of Upstate Biotechnology, Inc. (Lake Placid, NY). Briefly, the samples were sonicated to shear DNA to lengths between 200 and 1000 bps and then incubated for 16 hours at 4° C with mouse anti-MITF monoclonal antibody, rabbit anti-Histone H3 antibody and IgG (C5, Calbiochem, La Jolla, CA). Immune complexes were precipitated and the MITF-binding DNA was purified. The PCR was performed with primers flanking the putative MITF binding sites. The PCR product was analyzed by agarose gel electrophoresis. The immunoblot was performed as previously described [8]. Antibodies used for immunoblot were listed in Supplementary Table 9.

### Statistical analysis

Overall survival curves were calculated by the Kaplan–Meier analysis, and the difference between survival curves was tested by log-rank test. Each cutoff point for overall survival for definition of the high/low-MITF expression groups is listed in Supplementary Table 4 and Table 5. The univariate and multivariate Cox proportional hazards regression with covariates age, gender, cell types, stage, and *MITF* expression was performed to evaluate the prognostic abilities of variables. Student’s t test, and Fisher’s exact test were used to compare the difference between groups for continue or categorical data, respectively. All statistical analyses were done by SPSS (IBM, Chicago, IL) and SAS 9 (SAS Institute Inc., Cary, NC). All tests were two sided and p-value <0.05 was considered statistically significant.

## Supplementary Material

Supplementary Materials and Methods

Supplementary Figures

Supplementary Tables
