# Supplementary material for: MITF functions as a tumor suppressor in non-small cell lung cancer beyond the canonically oncogenic role
Source: Aging (Albany NY). 2020 Dec 3;13(1):646–74. doi: 10.18632/aging.202171 (PMC7835003; doi:10.18632/aging.202171)
Supplement: Supplementary Materials and Methods [file aging-13-202171-s001.pdf]

SUPPLEMENTARY MATERIALS

Supplementary Materials and Methods

Cell culture and transfection

The human lung adenocarcinoma cell lines, CL1-0, CL1-1, and CL1-5, in ascending order of invasive competence, were established in previously studies [1]. Cells were cultured in RPMI-1640 medium (Gibco, Life technologies, Carlsbad, CA) with 10% fetal bovine serum. Short hairpin RNA (shRNA) used in MITF (GenBank NM_000248) silencing studies was purchased from Open Biosystems (Huntsville, AL) [2]. The shRNA sequence targeting the human MITF gene is 5'-GCTAAAGTGATAGAAAGGCACCGCCTTACCCAAGTAAAGCAGTACCTTTCTACCACTTTAGC-3' (the underline sequence matches MITF open reading frame nucleotide 94-122). A scrambled shRNA (5'-TGCTGTTGACAGTGAGCGATCTCGCTTGGGCGAGAGTAAGTAGTGAAGCCACAGATGTACTTACTCTCGCCCAAGCGAGAGTGCCTACTGCCTCGGA-3') which does not match any known mammalian gene was used as the scramble control. CL1-0 cells were transfected with shRNAs using the Lipofectamine^{TM} 2000 (Life technologies, Carlsbad, CA) and selected with 2.5 µg/ml puromycin (Sigma, St Louis, MO) for stably MITF-silenced transfectants according to manufacturer's instructions. The full-length human *MITF-A* cDNA (GenBank NM_198159) was amplified from CL1-0 cells by RT-PCR and primers (forward primer: GCCATGCAGTCCGAATCGGG and reverse primer: ACAAGTGTGCTCCGTCTCTTCCA) and cloned into the constitutive mammalian expression vector pEF6/V5-His TOPO (Life technologies, Carlsbad, CA).

Real-time RT-PCR

The mRNA expression level of MITF was detected by qRT-PCR on ABI prism 7900 sequence detection system (Applied Biosystems, Branchburg, NJ), performed in accordance with the manufacturer instructions. For the SYBR Green method, the MITF primers used were the following: forward primer MITF-F: 5'-CCGGCATTTGTTGCTCAGA-3' and reverse primer MITF-R: 5'- AGACCCGTGGATGGAATAAGG-3' as well as the TATA box-binding protein (TBP) TBP-F: 5'-TTTTCTTGCTGCCAGTCTGGAC-3' and TBP-R: 5'-CACGAACCACGGCACTGA TT-3'. TBP was used as the internal control. For the TaqMan method, the sequences of customized MITF detection probes were as follows: MITF forward primer: 5'-CCGGCATTTGTTGCTCAGA-3', reverse primer: 5'-ACTTGAAATGCAGGCTC-3', and the probe sequence: 5'- ACTTGAAATGCAGGCTC-3'. The TBP detection probe (Assay ID: Hs00427621_m1, Applied Biosystems, Branchburg, NJ) was used as the internal control. Other primers for MITF target genes were listed in Table S1. All experiments were performed in triplicate.

Cell proliferation

Cells from each clonal line were seeded onto 96-well plates (3×103 cells/well). After culturing for various durations, cell proliferation was evaluated by thiazolyl bluetetrazolium bromide (MTT) assay according to the manufacturer`s protocol (Chemicon, Temecula, CA). Briefly, 10 µl of the MTT solution (5mg/mL) was added to each well, the cells were cultured for another 4 hours at 37° C, and 100 µl of DMSO was added to each well and mixed vigorously to solubilize colored crystals produced within the cells. The absorbance at 570 nm (630 nm as the reference) was measured by using a multi-well scanning spectrophotometer Victor3 (Perkin-Elmer, Boston, MA). Experiments were performed three times in triplicate.

Statistical analysis

Overall survival curves were calculated by the Kaplan–Meier analysis, and the difference between survival curves was tested by log-rank test. Each cutoff point for overall survival for definition of the high/low-MITF expression groups is listed in Supplementary Table 7 and Table 8. The univariate and multivariate Cox proportional hazards regression with covariates age, gender, cell types, stage, and *MITF* expression was performed to evaluate the prognostic abilities of variables. Student's t test, and Fisher's exact test were used to compare the difference between groups for continue or categorical data, respectively. All statistical analyses were done by SPSS (IBM, Chicago, IL) and SAS 9 (SAS Institute Inc., Cary, NC). All tests were two sided and p-value <0.05 was considered statistically significant.

REFERENCES

1. Chu YW, Yang PC, Yang SC, Shyu YC, Hendrix MJ, Wu R, Wu CW. Selection of invasive and metastatic subpopulations from a human lung adenocarcinoma cell line. Am J Respir Cell Mol Biol. 1997; 17:353–60. https://doi.org/10.1165/ajrcmb.17.3.2837 PMID:9308922

2. Paddison PJ, Silva JM, Conklin DS, Schlabach M, Li M, Aruleba S, Balija V, O'Shaughnessy A, Gnoj L, Scobie K,

Chang K, Westbrook T, Cleary M, et al. A resource for large-scale RNA-interference-based screens in mammals. Nature. 2004; 428:427–31. https://doi.org/10.1038/nature02370 PMID:15042091

3. Strub T, Giuliano S, Ye T, Bonet C, Keime C, Kobi D, Le Gras S, Cormont M, Ballotti R, Bertolotto C, Davidson I. Essential role of microphthalmia transcription factor for DNA replication, mitosis and genomic stability in melanoma. Oncogene. 2011; 30:2319–32. https://doi.org/10.1038/onc.2010.612 PMID:21258399
