# Supplementary material for: MITF functions as a tumor suppressor in non-small cell lung cancer beyond the canonically oncogenic role
Source: Aging (Albany NY). 2020 Dec 3;13(1):646–74. doi: 10.18632/aging.202171 (PMC7835003; doi:10.18632/aging.202171)
Supplement: Supplementary Figures [file aging-13-202171-s002.pdf]

SUPPLEMENTARY FIGURES

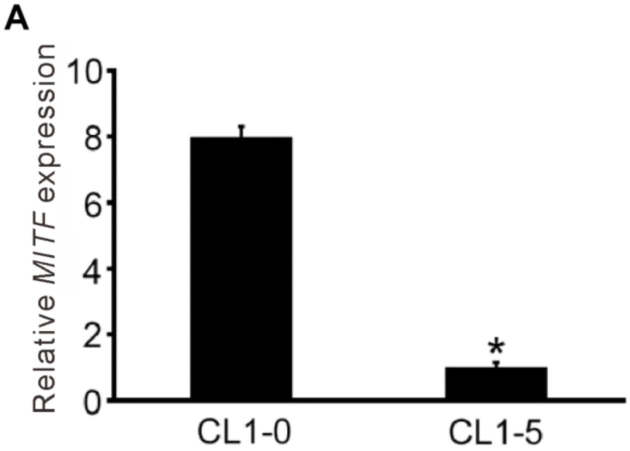

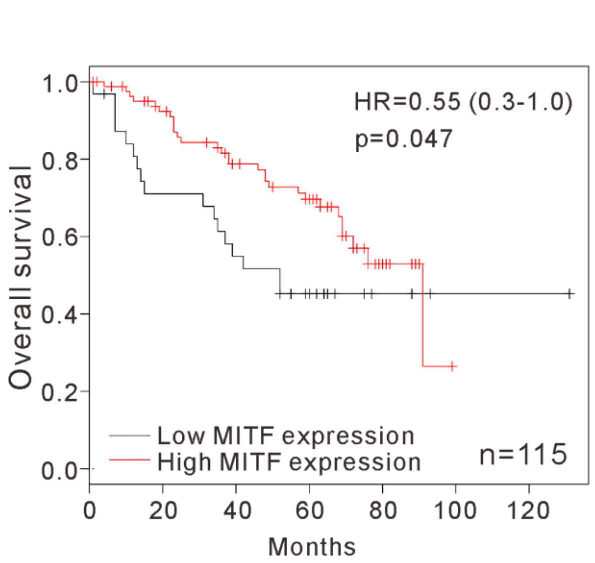

Multivariate analysis

	P value	Hazard Ratio
Stage	0.4891	1.87 (0.32 - 10.94)
AJCC stage T	0.0242	2.61 (1.13 - 6)
AJCC stage N	0.6275	1.56 (0.26 - 9.27)
Gender	0.2086	1.5 (0.8 - 2.82)
Smoking history	0.644	0.82 (0.35 - 1.9)
Selected gene	0.16	0.62 (0.32 - 1.21)

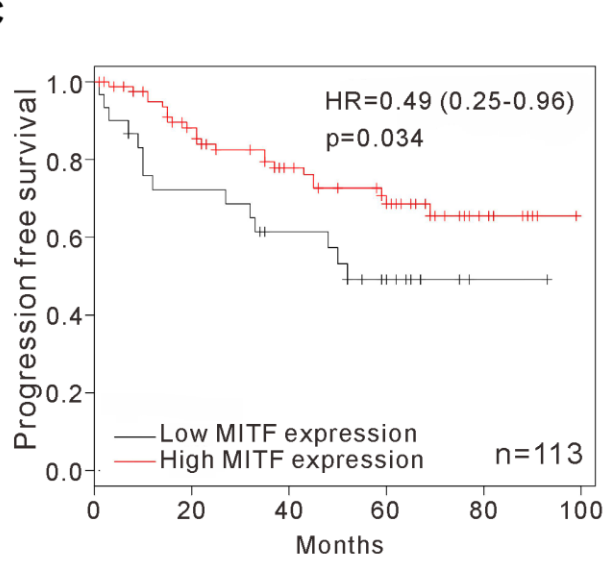

Multivariate analysis

	P value	Hazard Ratio
Stage	0.6569	0.58 (0.05 - 6.3)
AJCC stage T	0.0201	3.58 (1.22 - 10.52)
AJCC stage N	0.2207	4.41 (0.41 - 47.31)
Gender	0.4659	1.3 (0.64 - 2.61)
Smoking history	0.3132	1.52 (0.68 - 3.4)
Selected gene	0.0652	0.5 (0.24 - 1.04)

Supplementary Figure 1. *MITF* expression in an invasive cell line model and associated with survival in lung adenocarcinoma. (**A**) The relative *MITF* expression in low-invasive CL1-0 and high-invasive CL1-5 cells evaluated by expression microarray. (**B**, **C**) The survival curve of NSCLC was estimated with array probe of *MITF* (226066_at) by Kaplan-Meier plotter (https://kmplot.com/). The Hazard Ratio (HR) and p-value in the upper plots were estimated by univariate analysis and log-rank test, respectively. Lower panel listed the HR and p-value estimated by multivariate Cox's regression analysis.

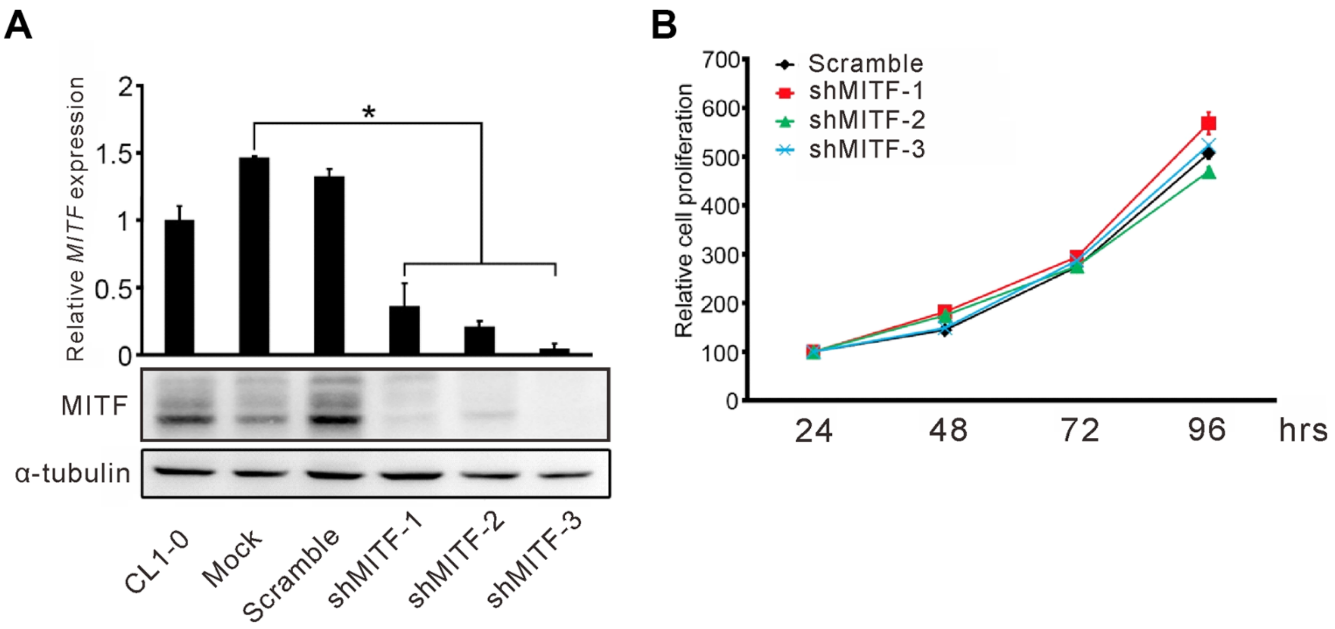

Supplementary Figure 2. The stable MITF-knockdown clones were assayed the cell proliferation. (**A**) Stable-expressed shMITF in CL1-0 cells were established in culture with puromycin. (**B**) The cell proliferation was analyzed by MTT assay at indicated time points.

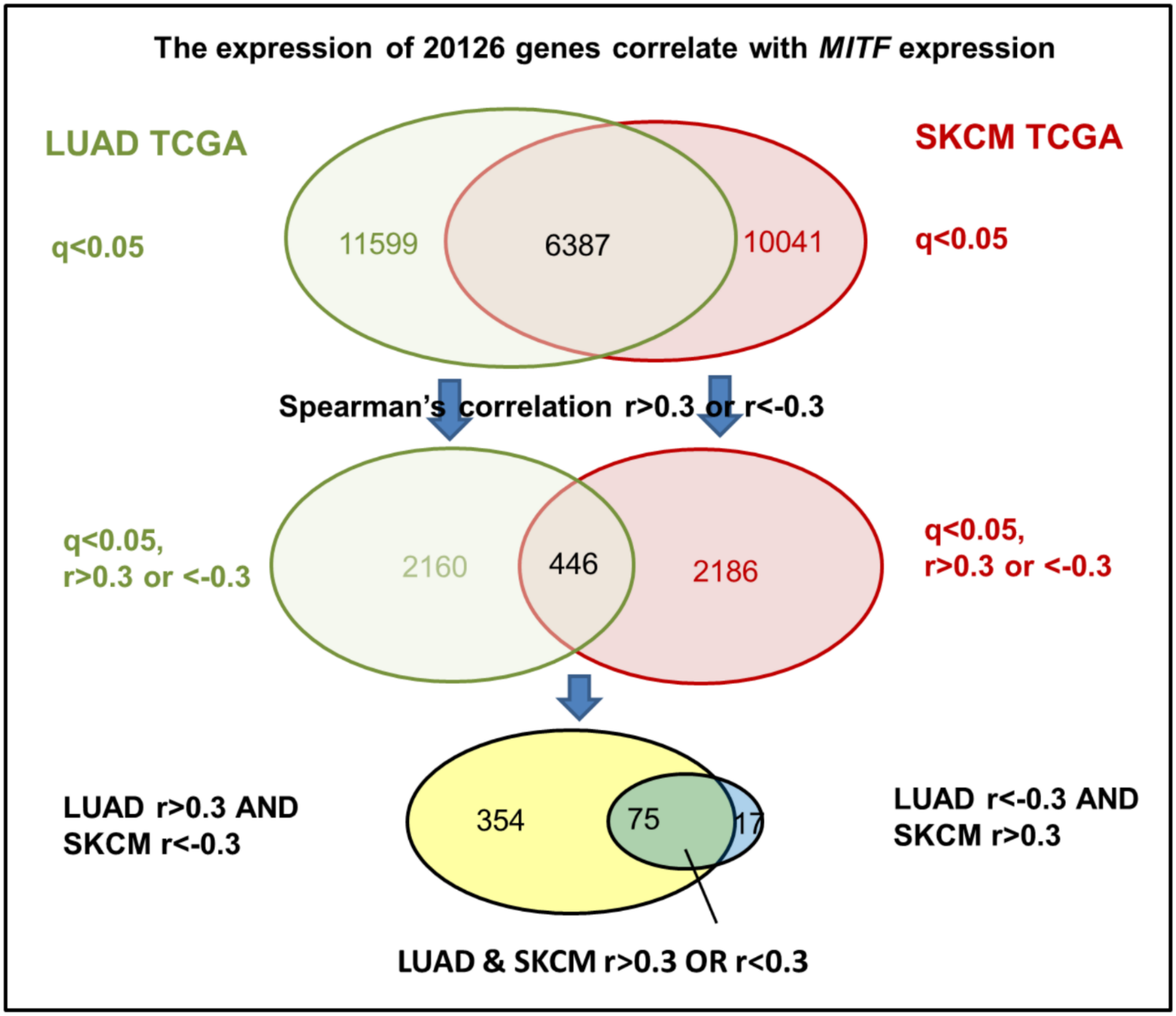

Supplementary Figure 3. The step-wise selection of Table 3. The spearman's correlations of MITF expression with 20,126 genes were sorted by cBioportal from the LUAD and SKCM TCGA datasets. The correlations with q<0.05 and r>0.3 or <-0.3 were filtered and intersected by the two datasets. Only 75 genes had the same expression directions along with MITF expression in LUAD and SKCM datasets. In contrast, 371 genes showed inverse correlations. The final data was presented in Table 3.

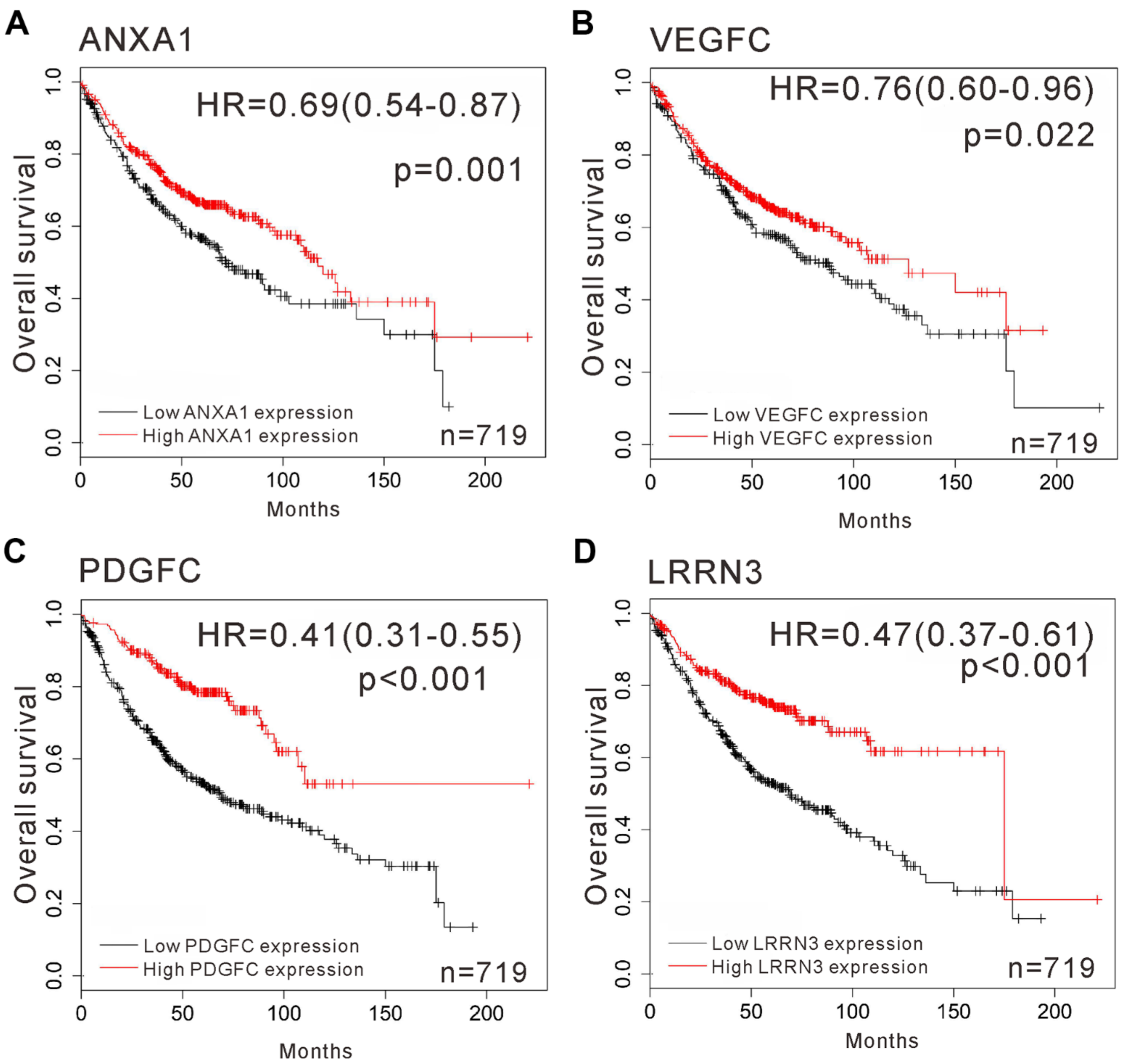

Supplementary Figure 4. The correlation of overall survival and *ANXA1*, *VEGFC, PDGFC* and *LRRN3* expressions in lung adenocarcinoma by Kaplan-Meier analysis. The available microarray databases of lung adenocarcinoma analyzed by Kaplan-Meier plotter (https://kmplot.com/). The probe (**A**) *ANXA1* (201012_at), (**B**) *VEGFC* (209946_at), (**C**) *PDGFC* (218718_at) and (**D**) *LRRN3* (209840_s_at). The p-value was estimated by log-rank teat. Univariate Cox's regression generated the hazard ratio.
