# Supplementary material for: MITF functions as a tumor suppressor in non-small cell lung cancer beyond the canonically oncogenic role
Source: Aging (Albany NY). 2020 Dec 3;13(1):646–74. doi: 10.18632/aging.202171 (PMC7835003; doi:10.18632/aging.202171)
Supplement: Supplementary Tables [file aging-13-202171-s003.pdf]

SUPPLEMENTARY TABLES

Supplementary Table 1. Characteristics of 70 NSCLC patients according to *MITF* expression.

Variables	High *MITF* expression	Low *MITF* expression	p-value
**Age, mean (±SD)**	69.07±9.29	65.88±10.95	0.19†
**Gender**			
Male	32 (91.43)	27 (77.14)	0.19‡
Female	3 (8.57)	8 (22.86)	
**Stage**			
I and II	23 (65.71)	18 (51.43)	0.33‡
III	12 (34.29)	17 (48.57)	
**Primary Tumor**			
T1 and T2	30 (85.71)	30 (85.71)	1.00‡
T3 and T4	5 (14.29)	5 (14.29)	
**Regional Lymph Nodes**			
N0	22 (62.86)	16 (45.71)	0.23‡
N1, N2, and N3	13 (37.14)	19 (54.29)	
**Cell Type**			
Adenocarcinoma	20 (57.14)	22 (62.86)	
Squamous cell carcinoma	11 (31.43)	8 (22.86)	0.74‡
Large cell carcinoma	3 (8.57)	4 (11.43)	
Mixed (small/large cell carcinoma)	0 (0.00)	1 (2.86)	
Bronchioloalveolar carcinoma	1 (2.86)	0 (0.00)	

†T test
‡Fisher's exact test

Supplementary Table 2. The enriched pathways in *MITF*-silenced cells by gene set enrichment analysis (GSEA).

Hallmark pathways	Size	ES	NES	NOM p-value	FDR q-value
**Down-regulation**					
Bile acid metabolism	109	-0.56	-1.45	0.024	0.41
IL2/STAT5 signaling	193	-0.50	-1.40	0.008	0.32
Wnt Beta-catenin signaling	41	-0.61	-1.38	0.061	0.25
Cholesterol homeostasis	71	-0.55	-1.35	0.061	0.24
Myogenesis	196	-0.47	-1.34	0.016	0.21
**Upregulation**					
Interferon-alpha response	92	0.74	1.81	0	0
E2F targets	190	0.56	1.51	0.002	0.07
Interferon-gamma response	194	0.54	1.45	0.006	0.09
Mitotic spindle	198	0.50	1.33	0.022	0.25
G2M checkpoint	185	0.49	1.29	0.054	0.32

Supplementary Table 3. Top 10 significantly enriched pathways of MITF by MetaCore analysis.

Pathway	FDR q-value
Gamma-secretase proteolytic targets	2.376E-05
Angiotensin II/ AGTR1 signaling via p38, ERK and PI3K	1.145E-04
Regulation of Beta-catenin activity	1.815E-04
Role of heterochromatin protein 1 (HP1) family in transcriptional silencing	1.815E-04
Gamma-Secretase regulation of cell development	1.815E-04
Angiotensin II/ AGTR1 signaling via JAK/STAT	5.292E-04
MAPK-mediated proliferation	6.296E-04
Alpha-2 adrenergic receptor regulation of ion channels	6.296E-04
Melanocyte development and pigmentation	8.655E-04
Thromboxane A2 signaling pathway	8.983E-04

Supplementary Table 4. Prognostic capability of MITF on the overall survival of melanoma patients from TCGA SKCM dataset.

Variable	Log-Rank P value	Hazard Ratio (95% CI)
10%	0.337	1.232 (0.805-1.884)
20%	0.442	1.139 (0.818-1.586)
30%	0.442	1.122 (0.837-1.504)
40%	0.194	1.200 (0.911-1.58)
50%	0.017 *	1.390 (1.062-1.819)
60%	0.058	1.298 (0.991-1.699)
70%	0.034 *	1.362 (1.024-1.812)
80%	0.215	1.228 (0.888-1.698)
90%	0.115	1.400 (0.921-2.127)

*P-value > 0.05 was considered statistically significant.

Supplementary Table 5. Prognostic capability of MITF on the overall survival of lung adenocarcinoma patients from TCGA LUAD dataset.

Variable	Log-Rank P value	Hazard Ratio (95% CI)
10%	0.228	0.730 (0.437-1.220)
20%	0.357	0.836 (0.570-1.225)
30%	0.039*	0.707 (0.508-0.984)
40%	0.251	0.838 (0.620-1.133)
50%	0.790	0.961 (0.718-1.286)
60%	0.863	0.974 (0.726-1.309)
70%	0.884	0.977 (0.713-1.339)
80%	0.783	0.951 (0.665-1.360)
90%	0.630	0.894 (0.567-1.410)

*P-value > 0.05 was considered statistically significant.

Supplementary Table 6. The expression of MITF downstream genes in lung adenocarcinoma and melanoma.

	Correlation with MITF expression*				Expression in MITF-silenced cells§			
	**LUAD**	**q-value**	**SKCM**	**q-value**	**CL1-0**	**p-value**	**501MEL#**	**p-value**
**ANXA1**	0.35	4.81E-15	-0.34	5.79E-10	-1.66	1.26E-06	1.98	2.16E-05
**VEGFC**	0.31	2.27E-11	-0.41	1.59E-14	-5.86	1.84E-05	0.93	0.35
**PDGFC**	0.40	7.61E-19	-0.48	1.91E-20	-5.31	1.66E-06	1.46	0.02
**LRRN3**	0.34	3.24E-14	-0.34	2.27E-10	-7.15	4.81E-06	3.10	2.91E-13

*Spearman's rank correlation coefficients were obtained from TCGA database by cBioPotal analysis. LUAD: lung adenocarcinoma. SKCM: skin cutaneous melanoma.
§Expression was log2 fold-change compared to control.
#Fold-change of gene expressions was obtained from siMITF and siLuc treated 501MEL human melanoma cells [3].

Supplementary Table 7. Primer sequences for real-time RT-PCR and *MITF* isoform-specific RT-PCR.

Gene	Primer sequence
**MITF targeting genes**	
ANXA1	F: TTCAATACCATCCTTACCACCAGAA
	R: CTCCAGGTCCAGAACTTTGTTCA
FZD7	F: CGCCTCTGTTCGTCTACCTCTT
	R: TTCTCCAGCTTCTCGGTCTTG
PTGR1	F: TCAGGAGCTTCGCATGGAA
	R: CCTCTAAGACCCATTTCAGCAAGT
**MITF 5'-primers**	
MITF-A	F: TGAAGAGCCCAAAACCTATTACGA
MITF-H	F: GGAGGCGCTTAGAGTTCAGATG
MITF-B	F: CCAAAGTGCAAACGAAGGGTCTCA
MITF-M	F: CCTTCTCTTTGCCAGTCCATCTTC
MITF-C	F: CTTCAGTGGTTTTCCCACGAGCT
MITF-E	F: AGTAGCAGGGGTTAGTAGGTGGAT
MITF-D	F: GTTTTAACCTGACAGGCTTTGAATA
MITF-J	F: CTCTCCATGAGTCTGAGCATCTAA
MITF-exon5	F: CCAGCCAACCTTCCCAACATAAAA
**MITF 3'-primers**	
MITF-com	R: GATCAATCAAGTTTCCCGAGACAG
MITF-exon9	R: CCGGGGGACACTGAGGAAAGGAG
GAPDH	F: GAAGGTGAAGGTCGGAGT
	R: GAAGATGGTGATGGGATTTC

Supplementary Table 8. Primers for chromatin immunoprecipitation assay of MITF.

Gene target*<br>(Reference GenBank ID)	Primer sequence	Location[#]	Amplified length<br>(bp)
ANXA1	F: TTAAGTATGTCCCTAAAGTCACA	-441→-419	338
(NM_000700)	R: TCAGCTATGTCCAAAAACAA	-123→-104	
FZD7-1	F: CTGGTTTCTAAGACGCATTA	-1792→-1773	201
(NM_003507)	R: TTGGGGAGACTGGTTCTA	-1609→-1592	
FZD7-2	F: AGCCTTTGCAGTCCTAAC	-1237→-1220	207
	R: GGAAAAACTTTTACCCTGAA	-1012→-1031	
PTGR1	F: CCTGCCTTTGGACCCTTC	-420→-403	289
(NM_012212)	R: GGAAATCTGAGTGCGTTTGTG	-152→-132	
GAPDH	F: AATTCCCCATCTCAGTCGT	-385→-367	233
(NM_002046)	R: CTACTTTCTCCCCGCTTTTT	-172→-153	

*The reference GenBank ID shows in the parenthesis.
[#]Nucleotide positions are numbered relative to the start site of transcription.

Supplementary Table 9. Antibodies for immunoblots.

Antibody	Clone	Dilution	Manufacturer	Cat No.
Anti-MITF	C17	1:100	Santa Cruz	SC-11002
Anti-MITF	C5	1:1000	Calbiochem	OP126L
Anti-FZD7		1:500	GeneTex	GTX64603
Anti-α-tubulin	DM1A	1:5000	Abcam	ab7291
Anti-V5		1:5000	Invitrogen	R960-25
